# Editorial

**DOI:** 10.1080/26415275.2021.2010366

**Published:** 2021-12-10

**Authors:** Anne Peutzfeldt, Jon E. Dahl

**Affiliations:** Editor-in-Chief Institute of Odontology, University of Copenhagen, Nørre Allé 20, 2200 Copenhagen, Denmark; Associate Editor Nordic Institute of Dental Materials, Oslo, Norway

Dear Reader,


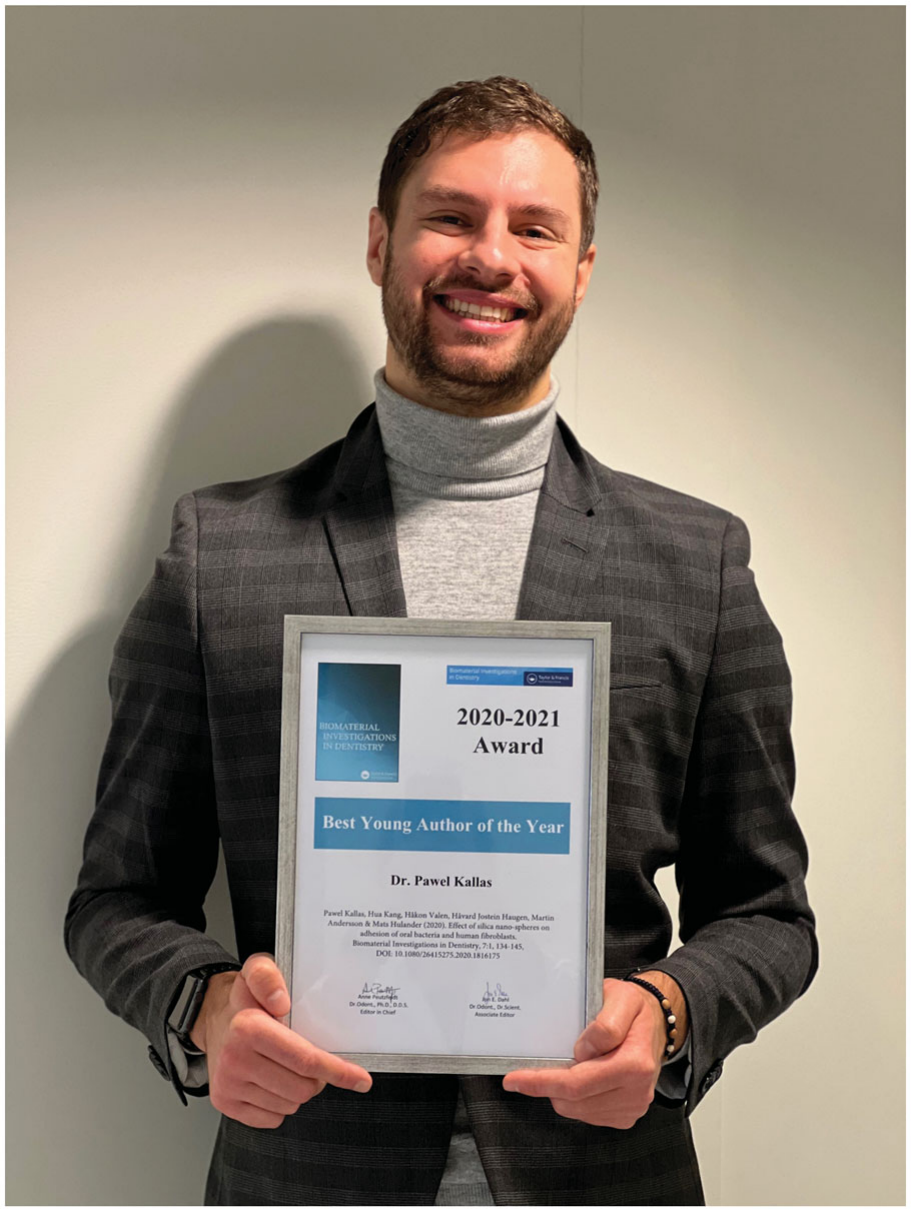


It is with great pleasure that we announce the recipient of the 2020–2021 Best Young Author of the Year Award: Dr. Pawel Kallas of the Institute of Clinical Dentistry, University of Oslo. Dr. Kallas receives the award for his paper: ‘Effect of silica nano-spheres on adhesion of oral bacteria and human fibroblasts’ published online on 15 September 2020. The paper, which is co-authored by Hua Kang, Håkon Valen, Håvard Jostein Haugen, Martin Andersson and Mats Hulander, was awarded for reporting on a study which aims to help tackle a growing clinical problem through an original approach and a thorough and convincing study design.

The award aims to encourage young scientists to publish their research in Biomaterial Investigations in Dentistry and to showcase what is a good manuscript. The award is presented to a first author who at the time of submission of his/her manuscript is within 10 years of completing his/her last terminal degree (PhD, DDS, DMD, MD, etc.), and who is of Nordic nationality or has conducted his/her research in a Nordic country (limited geographically by the statutes of the ACTA Odontologica Scandinavica Society). Eligible manuscripts are evaluated based on the following criteria: originality of study, suitability of study design and clarity of manuscript. The award is accompanied by a prize of 2.000 Euro and a diploma.

Our sincere congratulations to Dr. Kallas and his colleagues.

